# Post-fire soil hazards: recommendations for updated soil testing protocols and clearance thresholds

**DOI:** 10.1038/s41370-025-00796-w

**Published:** 2025-08-08

**Authors:** Joseph G. Allen, Parham Azimi, Gen Pei, Lauren Feguson, Lindsey Burghardt, Kari Nadeau

**Affiliations:** 1 Department of Environmental Health, Harvard T.H. Chan School of Public Health, Boston, MA USA; 2 Center on the Developing Child, Harvard University, Boston, MA USA; 3 Harvard Medical School, Boston, MA USA

**Keywords:** Child Exposure/Health, Personal Exposure, Wildfires, Metals

## Abstract

**Background:**

Urban wildfires in Los Angeles have highlighted the increased risk of soil lead exposure, especially for children. Current post-wildfire soil remediation protocols may not sufficiently protect public health, especially in communities returning after fire events.

**Objective:**

To evaluate the adequacy of existing soil remediation practices after urban wildfires in Los Angeles and present policy recommendations to reduce lead exposure risk.

**Methods:**

We reviewed current wildfire debris removal protocols, soil testing practices, and health risk benchmarks for lead exposure in California. We assessed recent data from post-fire soil testing and analyzed the scientific rationale underlying California’s existing Preliminary Remediation Goal (PRG) for lead in residential soil.

**Results:**

We recommend two critical reforms: requiring post-clearance confirmatory soil testing after wildfire cleanup, as has been done for every major wildfire in California since 2007, and lowering California’s residential Preliminary Remediation Goal (PRG) for lead in soil from 80 to 55 mg/kg to reflect updated science and health-protective standards. The basis for these recommendations is that repeated testing after purported soil remediation is showing that greater than 20% of properties still have lead levels that exceed existing thresholds, and the 80 mg/kg PRG (1) does not adhere to the health-based toxicity criterion benchmark set by California, (2) is susceptible to high uncertainty based on the values for several exposure factors used, and (3) does not accurately reflect our current understanding of risks to children from lead.

**Impact statement:**

- This article identifies critical gaps in current post-wildfire remediation protocols that leave Los Angeles residents, especially children, at risk of lead exposure from contaminated soil. By recommending policy reforms including mandatory post-remediation soil testing and a more protective soil lead standard, our work provides an actionable roadmap to strengthen environmental health protections for communities recovering from wildfires. Adoption of these measures will help ensure a safer, healthier future in the face of escalating urban wildfire threats.

## Introduction

Devastating urban fires in Los Angeles County in January 2025 have heightened public concern about lead contamination in residential soil. Urban fires burned through structures and materials containing lead such as paint and plumbing, as well as arsenic and other toxic metals [1]. While the immediate aftermath of wildfires understandably centers on loss of life and property, environmental health threats posed by legacy contaminants in soil can persist for years after flames are extinguished. Initial air monitoring after the LA Fires revealed an alarming 110-fold increase in atmospheric lead levels [2]. Soil testing later confirmed elevated lead levels in areas downwind of the Eaton fire [3]. This poses a long-term risk of exposure, particularly for biologically sensitive populations like children, through contact with contaminated soil or inhalation of lead dust.

Wildfires create particular risk by depositing ash laden with heavy metals and generating unpredictable “hotspots” of contamination that can be missed by standard debris removal. In California, post-wildfire soil testing following debris removal has been a standard practice in many previous major wildfire events, often serving as a critical step to ensure properties meet remediation standards [4]. However, for the Los Angeles fires in 2025, the Federal Emergency Management Agency and Army Corps of Engineers have only agreed to remove hazardous ash and up to a 6-inch layer of topsoil from destroyed properties and are not conducting post-remediation confirmatory testing of the soil [5].

Even low-level lead exposures can result in cognitive deficits, behavioral disorders, and other permanent harm in children, for whom there is no known “safe” level of lead [6]. Given the well-documented risks of lead exposure to children’s neurodevelopment and lifelong health [7], and the well-documented contamination of soil after urban wildfires, it is imperative that policymakers modernize soil clearance protocols to ensure a safe return for families and future generations.

## Public health recommendations

In this Perspective, we advance two urgent and evidence-based recommendations to strengthen public health protection and community resilience in Los Angeles and other wildfire-affected regions: (1) mandating confirmatory soil testing following wildfire debris clearance, and (2) reducing the PRG for lead in residential soil from 80 mg/kg to 55 mg/kg.

## Recommendation 1

### Require post-clearance soil testing for all burned properties

Testing of soil after debris removal has been the norm in California. However, for the fires in L.A. in 2025, Federal Emergency Management Agency and Army Corps of Engineers have only agreed to remove hazardous ash and up to a 6-inch layer of topsoil from destroyed properties and are not doing post-remediation confirmatory testing of the soil [8].

### Soil removal without testing to verify is insufficient

Wildfires in California have repeatedly shown that debris removal alone does not guarantee that residential properties are free from hazardous levels of soil contamination. Since 2017, concerns have been raised by state and local officials, as well as academic researchers, that a significant percentage of residential properties tested after wildfire cleanup still contained contaminants, such as lead and arsenic, that exceed health-risk thresholds [9]. The Los Angeles Times reported on May 4, 2025 that 20% of Army Corps-remediated homesites in Altadena still exceeded lead safety standards [10]. Testing conducted by the L.A. County Department of Public Health similarly found that 27% of cleaned lots still had unsafe lead levels in soil [11]. Prior wildfires have also revealed contamination from other metals such as arsenic, cobalt, mercury, and zinc [12]. Without parcel-specific confirmation testing, homeowners and regulators have no way to verify a property has been successfully remediated—posing risks to residents, contractors, and financial institutions. Moreover, the absence of thorough testing impairs the detection of potential fraud or contractor under- or over-excavation during debris removal.

#### The precedent: ventura county’s model

There is already an established soil sampling and debris removal protocol that was used in Ventura County [8]. This approach, used successfully after the 2017 Thomas Fire, 2018 Woolsey Fire, and 2024 Mountain View Fire, integrates soil confirmation testing into debris removal contracts backed by CalOES and CalRecycle. Properties that participated received detailed soil testing reports and official completion documentation, enabling them to move forward confidently with rebuilding.

The absence of confirmatory testing fails residents and rebuilders—not only by exposing them to invisible health hazards, but also by undermining trust in public recovery programs. Without parcel-specific data, residents, contractors, insurers, lenders, and financial institutions cannot reliably certify that a property is safe. Furthermore, without monitoring, there is no safeguard against fraudulent or incomplete remediation, nor a method to assess under- or over-excavation that can incur unnecessary ecological and financial costs.

#### Recommendation: adopt ventura county’s model for addressing hazards in soil after urban fires

We also urge Los Angeles County (and all California fire response programs) to adopt the Ventura County model:

- Integrate post-clearance soil confirmation testing into all debris removal contracts.
- Require testing for both lead and other toxic metals (e.g., arsenic, mercury).
- Provide publicly accessible test results and certification for homeowners and property buyers.

## Recommendation 2

### Lower the residential soil lead preliminary remediation goal (PRG) from 80 mg/kg to 55 mg/kg

California’s Office of Environmental Health Hazard Assessment established a health-based benchmark of a 1.0 µg/dL incremental increase in children’s blood lead levels (BLLs), associated with an average loss of one IQ point, as the basis for establishing protective measures related to lead in soil [13]. This benchmark was used by California’s Department of Toxic Substances Control’s (DTSC) “LeadSpread9” model to create a corresponding Preliminary Remediation Goal (PRG) for lead in soil of 80 mg/kg [14].

DTSC’s LeadSpread 9 is the model used to estimate the residential lead soil screening level. The U.S. EPA’s Adult Lead Model (ALM) used the DTSC’s LeadSpread 9 model to estimate the blood lead concentration in a fetus of an adult worker exposed to lead-contaminated soil. This is the concentration that would correspond to an estimated increase in blood lead in a 90^th^ percentile child of 1 µg/dL. The model reflects four exposure pathways: (1) incidental ingestion of outdoor soil and, (2) incidental dust ingestion from indoor dust from soil tracked into a home, (3) dermal uptake from contact with outdoor soil or indoor dust, and (4) inhalation of resuspended particles. In the current model, ingestion is the dominant pathway; there is little contribution from inhalation of resuspended soil and dust and dermal uptake of lead. Thus, the choice of exposure factors for dust ingestion has a large influence on the resulting BLL calculation. DTSC uses a soil+dust ingestion rate of 80 ug/day for children, based on the central tendency from the EPA Exposure Factors Handbook.

#### Issue 1: misalignment with California’s health-protective benchmark

DTSC acknowledges that they use the toxicity criterion from OEHHA of 1.0 ug/dL rise in children’s blood lead levels as the basis for their approach at setting soil limits for lead:

*“The toxicity criterion on which LeadSpread 9 is based is CalEPA’s Office of Environmental Health Hazard Assessment’s (OEHHA) toxicity evaluation of lead with a source-specific “benchmark change” of 1 µg/dL which is the estimated incremental increase in children’s blood lead that would reduce IQ by up to 1 point.”*

However, in their calculations using LeadSpread9, DTSC acknowledges that **70 mg/kg** is the soil PRG that is estimated using their tool:

*“Using the previous version of LeadSpread, LeadSpread 8, a Preliminary Remedial Goal of 77 mg/kg soil lead was estimated. A value of 70 mg/kg soil lead is estimated using LeadSpread 9.”*

Despite this, DTSC attempts to explain that 80 mg/kg should be used, even though it is actually based on a rise in BLL of 1.14 μg/dL, not the 1.0 μg/dL benchmark from OEHHA:

*“For most sites without special circumstances, such as markedly elevated soil lead bioavailability, the difference in predicted incremental blood lead and IQ change for exposures to soil lead between 70 mg/kg and 77 mg/kg is within the LeadSpread model uncertainty and does not exceed the de minimis level of 1 IQ point identified by OEHHA. The current DTSC residential lead (Pb) soil screening level is 80 mg/kg, based on an estimated increase in blood Pb in a 90*^*th*^ *percentile child of 1 µg/dL. At 80 mg/kg soil lead, LeadSpread 9, estimates the increase in blood Pb in a 90*^*th*^ *percentile child as 1.14 µg/dL which, in turn, is associated with an upper-bound estimate of a loss of 1 IQ point. The change is not discernable at one significant figure. Results of IQ tests are reported as an integer. Fractional IQ points are not measured. The blood lead level of 1.14 would have to rise to 1.5 (which would round up to 2.0) to be considered a significant increase. Therefore, HERO recommends that the remedial/mitigation level for residential soil exposure remain at the current residential default value of 80 mg/kg. Future development of better-defined childhood exposure parameters may change this recommendation.”*

We disagree that the rounding is inconsequential and there is not a strong basis for DTSC to depart from the OEHHA benchmark of 1.0 μg/dL. Using OEHHA’s benchmark of 1.0 μg/dL, the soil lead PRG should be 70 mg/kg, with no other calculations changed.

#### Issue #2: uncertainty with EPA’s exposure factors used by DTSC

DTSC’s LeadSpread9 model uses several exposure factors to estimate the amount of lead in soil that would lead to the corresponding BLL. Here, we demonstrate the high level of uncertainty for one exposure factor, “ingestion constant”, as an example of how assumptions for these factors can lead to significantly different PRGs.

DTSC uses an ingestion contact of 0.16 (µg/dL)/(µg/day) in LeadSpread9. This exposure factor is ratio of blood level to lead that enters the body through the ingestion pathway, essentially capturing the fraction of lead that contributes to a rise in blood lead. There are several issues with this ingestion constant: it is from an old study conducted in the early 1980s [15], it is not technically scientifically accurate because it uses liquid ingestion and extrapolates to soil ingestion, and it has a small sample size, all of which indicate high uncertainty in this ingestion constant.

The estimate for the ingestion contact originates from a 1983 study of 29 breast-fed and formula-fed infants [15]. The authors measured the amount of lead the infants consumed in milk and the corresponding increase in BLLs. In addition to the small sample size and the age of the study, a critical issue is the assumption that this constant, derived from the ingestion of formula and breast milk, also applies to the ingestion of soil and dust. The original study included a high- and a low-exposure group, which were combined to calculate the 0.16 (µg/dL)/(µg/day) constant. However, if the data from each exposure group and age category are used separately, this value could be different. For example, among infants aged 112–195 days who remained in the study, the ingestion contact was approximately 0.2 or 0.4. If the LeadSpread9 model is used with this constant adjusted to either 0.2 or 0.4, the resulting PRG changes from 70 to 56 mg/kg and 28 mg/kg, respectively.

Our main goal of mentioning this study is less about opening a debate about which constant to use from this study in 1983; rather, we are using it to show how sensitive the model is to exposure assumptions.

#### Issue #3: children are more susceptible to lead than the current model accounts for

The substantial advances in developmental science since the original model was developed suggest that LeadSpread 9 model likely underestimates the effects of lead under current exposure estimates, both in the adult models that account for sensitivity to the fetus and the model for childhood, which accounts for lead only as a single exposure (which in reality, never occurs in young children). They also fail to account for differential sensitivity to lead across childhood and assumes the effects on children ages 1–6 years of age, despite these ages having very different behaviors and sensitivities to exposure [16].

Both adult models (residential and industrial) account for fetal sensitivity to lead use the same standard that is used in infants, of protecting the fetus carried by an exposed adult to prevent an increase in blood lead of the fetus of >1 µg/dL. This logic is in direct opposition to the abundance of studies showing that fetal sensitivity of both the brain and other rapidly developing biological systems is greater than the sensitivity of infants on whom the original model was based [17]. The half-life of lead is also increased during pregnancy, leading to longer exposure times for the same dose of exposure [18]. Given this differential sensitivity, it is nearly a guarantee this same blood lead level increase in a fetus would have greater adverse effects than the same level rise in an infant of older child. Additionally, blood lead levels in the fetus have been identified simultaneously as higher than in the mother [19].

The risk of compounding environmental exposures, which are now felt to be critical in understanding and calculating risks to fetal and early childhood development, are not accounted for at all in the original model. For example, blood lead levels can increase more rapidly in children with iron deficiency anemia, a condition that is likely to worsen when children are displaced and have less consistent access to mitigating factors like iron-rich foods [20]. Science increasingly demonstrates that co-exposure to psychosocial stress, which is significantly increased following wildfire events, and lead increase the harmful effects of each of these exposures alone [21]. Because their brains and biological systems are so rapidly developing, children are especially sensitive to both direct psychosocial stress as well as the indirect stress experienced by their caregivers.

Furthermore, the model’s scope is confined to acute toxicity and does not adequately account for the systemic health consequences of chronic lead exposure. Substantial scientific evidence has demonstrated that lead negatively impacts multiple organ systems beyond the nervous system, including the cardiovascular system [22], renal function [23], endocrine signaling [24], and the immune system [25]. By neglecting these broader health effects, the current model fails to provide a comprehensive risk assessment, ultimately undermining efforts to fully protect vulnerable populations from the multifaceted harms of lead exposure.

Finally, both lead ingestion and lead absorption change throughout the first two years of life. Assuming that the model holds for children ages 1–6 is especially problematic given how different children are even within that range. For children consistently exposed to lead, blood levels increase rapidly between 6 and 12 months of age, are highest form 18 months to 36 months of age, and then decrease gradually [7]. After that time, dust ingestion from ‘hand to mouth’ behaviors become the dominant exposure route as children become more mobile.

#### Recommendation: adjust soil screening level from 80 mg/kg to 55 mg/kg

To account for DTSC’s misalignment with toxicity criterion from OEHHA, high levels of uncertainty in DTSC’s LeadSpread9 model, and to capture the current state of science with regard to the impacts of lead on children’s health, we recommend using a PRG of 55 mg/kg. The basis for this updated PRG is the following:

- Re-aligning with OEHHA’s toxicity criterion and using OEHHA’s benchmark of 1.0 µg/dL BLL increase as the *de minimis* threshold.
- Accounting for parameter uncertainty and updated science on kids health and using the high-end central tendency for children’s soil ingestion of 100 mg/day from the EPA Exposure Factors Handbook, rather than the 80 mg/day used by DTSC.

When using these parameters, without changing any other default values or calculations used by DTSC in LeadSpread9, the resulting PRG is 56 mg/kg, and we use 55 mg/kg for simplicity.

#### Other key considerations:

We note the following:

- A PRG of 55 mg/kg is a soil remediation level below which no further action is needed on the site for full use; levels above this should be remediated.
- This PRG does not account for other potential exposures in a residence. If there are other sources, a site-specific risk assessment may be warranted, as noted by DTSC:

*“Because the lead benchmark dose is an incremental change in blood lead, background exposures to lead, and media other than soil, or dust from the site which may be impacted by lead are not considered in the worksheet. If lead is present in media other than soil (e.g., water, air) or if the home grown produce pathway is anticipated at the site, please contact the HERO toxicologist assigned to the site.”*

- The exposure assumptions are for exposed soil; if there is ground cover, or if there is fresh topsoil, exposure will be lower.
- In addition to remediating soils, we recommend the following individual actions that can help reduce exposure:
  - Wash hands frequently, especially before eating
  - Remove shoes when entering a residence
  - Clean the paws of pets before entering a residence
  - Keep indoor surfaces clean
  - Damp wipe dirty surfaces, especially playroom floors, carpets, and foam or rubber mats, where children play on them more often
  - Use a vacuum with a HEPA filter

- These values are intentionally designed to protect children; adults who have less exposure to soil will have lower risk.

## Conclusion: a call to policy action

The January 2025 urban fires in Los Angeles mark a critical juncture for public health practice in wildfire recovery, highlighting the different pollutant mix from urban fires compared to wildland fires, and the persisting hazards of metals. Scientific evidence, regulatory precedent, and the well-established developmental risks of childhood lead exposure all converge on the urgent need for reform in soil remediation policy. Without confirmatory soil testing after debris removal, residents and rebuilders are potentially left vulnerable to invisible but potent neurotoxicants, undermining both individual health and trust in recovery efforts. Furthermore, the current soil lead Preliminary Remediation Goal (PRG) of 80 mg/kg does not reflect updated scientific understanding of lead toxicity, children’s unique susceptibility, or the significant uncertainties in prevailing exposure models.

By adopting post-clearance soil confirmation testing and lowering the PRG for lead in residential soil to 55 mg/kg, Los Angeles County and other fire-impacted jurisdictions can ensure that updated, evidence-based, health-protective standards are being used. This dual approach not only aligns cleanup with best practices and the most current science on children’s health but also restores public confidence and supports the safe return of families. In the face of an increasing wildfire risk, including those in urban landscapes, public agencies must update protocols to close critical gaps in environmental health protection. The long-term safety, cognitive development, and well-being of the next generation depend on our resolve to fully confront the lingering threat of lead in post-wildfire communities.
